# Youth with Down syndrome display widespread increased functional connectivity during rest

**DOI:** 10.1038/s41598-022-13437-1

**Published:** 2022-06-14

**Authors:** Kelsey D. Csumitta, Stephen J. Gotts, Liv S. Clasen, Alex Martin, Nancy Raitano Lee

**Affiliations:** 1 Department of Psychological and Brain Sciences, Drexel University, Philadelphia, PA 19103 USA; 2 Section on Cognitive Neuropsychology, Laboratory of Brain and Cognition, National Institute of Mental Health, National Institutes of Health, Bethesda, MD USA; 3 Section on Developmental Neurogenomics, Human Genetics Branch, National Institute of Mental Health, National Institutes of Health, Bethesda, MD USA

**Keywords:** Human behaviour, Cognitive neuroscience

## Abstract

Studies of resting-state functional connectivity in young people with Down syndrome (DS) have yielded conflicting results. Some studies have found increased connectivity while others have found a mix of increased and decreased connectivity. No studies have examined whole-brain connectivity at the voxel level in youth with DS during an eyes-open resting-state design. Additionally, no studies have examined the relationship between connectivity and network selectivity in youth with DS. Thus, the current study sought to fill this gap in the literature. Nineteen youth with DS (*M*_age_ = 16.5; range 7–23; 13 F) and 33 typically developing (TD) youth (*M*_age_ = 17.5; range 6–24; 18 F), matched on age and sex, completed a 5.25-min eyes-open resting-state fMRI scan. Whole-brain functional connectivity (average Pearson correlation of each voxel with every other voxel) was calculated for each individual and compared between groups. Network selectivity was then calculated and correlated with functional connectivity for the DS group. Results revealed that whole-brain functional connectivity was significantly higher in youth with DS compared to TD controls in widespread regions throughout the brain. Additionally, participants with DS had significantly reduced network selectivity compared to TD peers, and selectivity was significantly related to connectivity in all participants. Exploratory behavioral analyses revealed that regions showing increased connectivity in DS predicted Verbal IQ, suggesting differences in connectivity may be related to verbal abilities. These results indicate that network organization is disrupted in youth with DS such that disparate networks are overly connected and less selective, suggesting a potential target for clinical interventions.

## Introduction

Down syndrome (DS) is the most common genetic cause of intellectual disability, impacting approximately one in every 691 live births^1^. In addition to intellectual disability, the DS neuropsychological phenotype is characterized by specific neurocognitive impairments, especially in the domains of language (for review, see^2^) and executive functioning (e.g.^3–5^). However, the neural bases of cognitive challenges in this population have not been well-explored. Non-invasive neuroimaging techniques, such as functional magnetic resonance imaging (fMRI), offer an opportunity to elucidate the biological underpinnings of the learning challenges associated with DS and may be an important tool for identifying clinical interventions. Such research in young individuals with DS is of particular importance, as earlier interventions may help alter developmental trajectories to promote optimal outcomes. Moreover, as DS is a disorder characterized not only by neuro*developmental* atypicalities but also by precocious-onset neuro*degeneration* due to dementia^6^, it is crucial to focus research on youth and young adults with DS (i.e., at developmental stages well before the onset of dementia) in order to describe developmental brain states prior to the occurrence of neurodegenerative disease.

Most neuroimaging studies of individuals with DS have focused on brain structure. Volumetric studies (e.g.^7–12^) have indicated reduced total and regional brain volume (e.g.^7–14^), with prominent abnormalities in the frontal (e.g.^9,11,12^) and temporal (e.g.^8,9,11,12^) lobes. In addition, a study examining cortical thickness and surface area in youth with DS relative to TD controls, revealed decreased surface area in the frontal and lateral temporal lobes as well as increased cortical thickness in the frontal lobes^9^. Regions of the frontal and temporal lobes are thought to serve as hubs in network conceptualizations of the neural underpinnings of executive functioning^15^ and language^16^, and thus, may be of particular importance in understanding the DS neurocognitive phenotype given the notable impairments in these cognitive domains^2–5^. In addition to these neocortical abnormalities, reduced regional brain volume of the hippocampus (e.g.^14,17^) has been found using high-resolution imaging. Similarly, volumetric and diffusion tensor imaging (DTI) investigations have documented reductions in the volume of the cerebellum in individuals with DS (e.g.^12,18,19^). Of note, recent research has highlighted significant hypoplasia of frontal-pontine-cerebellar networks in individuals with DS, underscoring the importance of studying cortical-subcortical networks using functional neuroimaging^19^.

In spite of this, few studies to date have examined brain function in individuals with DS using fMRI (for review, see^20^). Of these, task-based functional imaging studies have examined verbal and visual-spatial abilities^21^, language processing^22,23^, and silent object naming^24^. These studies have documented hyper-activity in a variety of regions in this population. For example, relative to neurotypical peers, greater activation of frontal regions was found during tasks of language processing^22^ and silent object naming^24^, and greater precuneus and cingulate activity was found during language processing^23^. Additionally, greater activation was found in the superior temporal gyrus during silent naming^24^. Increased middle frontal gyrus and left parietal activations were found during verbal and visual-spatial tasks relative to controls^21^. In contrast to this pattern of hyper-activation in individuals with DS, a few studies have found reduced activation in temporal regions during language processing relative to controls^22,23^ and reduced occipital, parietal, and left frontal activity during verbal and visual-spatial processing^21^.

In contrast to measuring specific cognitive processes, resting-state paradigms measure spontaneous, slowly-fluctuating brain activity in the absence of external stimuli. This design allows for the measurement of “baseline” functional connectivity, or regions displaying synchronized fluctuations in activity over time (see^25,26^ for review). Very few studies have examined functional connectivity in individuals with DS using resting-state or quasi-resting-state study designs^27–30^. About half of these studies have focused on middle-aged to older adults with DS and about half have focused on young adults. We will begin with a review of the literature on studies of middle-aged to older adults and then turn our attention to studies of youth and young adults, the focus of the current research.

Resting-state studies that have included middle-aged adults with DS have tended to focus on the default mode network (DMN)^30,31^ or select, predefined networks^29^. Regarding the DMN, researchers have found a mix of increased and decreased connectivity in adults with DS relative to controls. Whereas Wilson^30^ found generally widespread increased connectivity (apart from a few localized regions of decrease), Rosas^31^ found a mix of increased connectivity (e.g., in frontal DMN regions) and decreased connectivity in other components of the DMN. Beyond the DMN, one study of 10 individuals with DS conducted by Vega^29^ investigated 21 pairs of predefined networks and revealed increased connectivity across six of the 21 network pairs examined relative to healthy adults. These findings suggest there may be a mix of hypo- and hyper-connectivity by network in adults with DS. However, the age of the participants studied and the heightened risk for early neuropathological signs of dementia in DS make it difficult to ascertain which findings relate to atypical neurodevelopment and which relate to age-related neurodegeneration. More specifically, as these studies included middle-aged and older individuals with DS (Wilson^30^: Age range 29–49 years; Rosas^31^: 40 years and above; Vega^29^: 30–55 years) and virtually all individuals with DS will present with the neurofibrillary plaques and tangles characteristic of dementia by 40 years of age^32^, these studies are limited in their ability to describe the neurodevelopmental phenotype in DS.

In order to understand this phenotype in DS to inform early intervention research, it is crucial to focus neuroimaging investigations on young samples, well before concerns about neurodegeneration exist. The current study seeks to fill this gap in the literature, as there have been exceptionally few studies to date that have examined resting-state activity in youth or young adults with DS. The first of these studies, conducted by Anderson^28^, utilized a quasi-resting-state design. They compared between-network connectivity across seven brain networks defined by Yeo^33^ while participants with DS or typical development (TD) watched cartoon videos. The DS group displayed increased connectivity across all networks examined (14 of 21 surviving multiple comparison correction). Additionally, a negative correlation was observed between connectivity and Performance IQ in DS. An ROI analysis also revealed increased correlations for short-range connections and those typically showing anticorrelations in controls, with a few exceptions for distal regions showing reduced correlation in DS. The second study, conducted by Pujol^27^, examined differences in whole-brain connectivity and regional connectivity during eyes-closed rest between individuals with DS and age- and sex-matched TD peers. In contrast to Anderson^28^, they found higher regional connectivity in some regions (e.g., amygdala/anterior temporal, anterior cingulate, frontal cortices) and lower connectivity in others (e.g., dorsal prefrontal, anterior cingulate cortex, posterior insula), with variations in connectivity relating to communication skills. Specifically, poorer communication skills were associated with increased connectivity in the ventral-medial frontal cortex and amygdala and reduced connectivity in the left posterior insula and right sensorimotor cortex^27^. A third study conducted by Figueroa-Jimenez^34^ investigated resting-state connectivity of the DMN in 22 young adults with DS (*M* age = 25 years, range 16–35 years) and 22 healthy control participants. This study revealed increased connectivity in ventral, sensorimotor, and visual DMN regions in the DS group relative to the control group. Fourth, Koenig^35^ investigated resting-state within-network connectivity of the DMN (measured from the cingulate cortex) in 11 young adults with DS (*M* age = 29 years, range 26–35 years) and 11 age- and sex-matched controls. They^35^ found increased connectivity between the anterior cingulate and the bilateral inferior frontal gyri and right putamen. In contrast, 68% of DMN connections to the posterior cingulate and 26% of DMN connections to the anterior cingulate revealed reduced connectivity in the DS group relative to the control group. Lastly, Koenig^17^ conducted a resting-state (eyes closed) functional connectivity study of the hippocampus and DMN in 22 teenagers and adults with DS (*M* age = 24.5 years, range 15–35 years) relative to neurotypical age-matched controls which revealed largely reduced hippocampi-DMN connectivity in the DS group.

The studies by Anderson^28^, Pujol^27^, Figueroa-Jimenez^34^, and Koenig^17,35^ provide an important foundation for understanding network connectivity in the developing brain in individuals with DS. However, they are characterized by the following limitations: (a) the use of a quasi-resting-state design^28^; (b) a focus on predefined networks rather than the whole brain^17,28,34,35^; and (c) the use of global signal regression or a similar normalization step^17,27,35^, which has been shown to alter group differences both quantitatively and qualitatively in prior research (see^36–42^ for discussion). In addition, these studies did not address two important, open questions in the field: (a) are differences in connectivity in DS network specific or diffuse, and (b) how does the degree of connectivity observed in DS relate to the network selectivity?

To address these limitations and help fill this gap in the literature, the current study sought to examine differences in whole brain functional connectivity acquired during eyes open rest between youth with DS (*M*_age_ = 16.53; range 7–23) and TD controls (*M*_age_ = 17.53; range 9–24) at ages well before the onset of Alzheimer’s dementia in order to further characterize the developing brain in this understudied population. In particular, the current study sought to answer the following three questions: (1) are there differences in resting connectivity in youth with DS compared to TD controls when examined at the voxel-level (without the implementation of global signal regression); (2) if differences in connectivity are observed across groups, are differences network-specific or diffuse; and (3) is connectivity related to network selectivity in DS.

## Methods

Participants were recruited as part of a larger neuroimaging study on individuals with DS^9^ conducted at the NIH Clinical Center. This study was approved by the Combined Neuroscience Institutional Review Board (clinical trials number NCT00001246), and all work described here was performed in accordance with relevant guidelines and regulations. Parental consent and child assent were obtained for legal minors. For adults with DS, capacity to provide informed consent was evaluated. If deemed capable, the participant provided informed consent. If not deemed capable of independent consent, a legally authorized representative provided informed consent and the adult participant provided assent. After completion of informed consent and assent procedures, participants completed cognitive testing as well as T1, DTI, and resting-state neuroimaging.

### Participants and procedures

Participants with DS were recruited from a larger study of brain and cognitive development (see^9^ for details). For this study, all participants were required to be (a) between the ages of 5 and 25 years, and (b) free of any acquired brain injuries or conditions which cause gross brain abnormalities. One exception was a participant with well-controlled Lennox-Gastaut syndrome. As epilepsy occurs in the DS population at higher rates^43^, we chose to include this individual in analyses. For participants with DS, a diagnosis of Trisomy 21 was required. TD participants were required to be free of any cognitive, behavioral, or neurological conditions in order to be included.

Of the participants from the larger study, 26 with DS and 38 TD participants had resting-state data. To be included in the current study, participants were required to have motion values < 0.30 mm/TR during resting-state scans (AFNI’s @1dDiffMag; mean frame-wise displacement). This resulted in the exclusion of 7 participants with DS and 5 TD participants. Participants with DS with usable scan data included in the current sample did not differ from those without usable (or any) scan data on intellectual ability level. However, younger age and being male was associated with being excluded (Supplementary Information Table S1).

Participants in the present study included 19 youth with a diagnosis of DS (*M*_age_: 16.53 years ± 4.99; range 7–23 years) and 33 TD youth (*M*_age_: 17.53 years ± 5.15; range 9–24 years). The majority of participants completed measures of Full Scale IQ (FSIQ), Verbal IQ (VIQ), and Performance IQ (PIQ) using either the Kaufman Brief Intelligence Test (KBIT, DS: *n* = 10, TD: *n* = 13) or the Differential Ability Scales (DAS, DS: *n* = 9, TD: *n* = 13), depending on the age-appropriateness of the measure. To increase power, seven additional TD participants who completed the same imaging procedures but different measures of intellectual functioning [Wechsler Abbreviated Scale of Intelligence (WASI, TD: *n* = 6), or the Wechsler Preschool and Primary Scale of Intelligence, Third Edition (WPPSI-III, TD: *n* = 1)] were also included in the sample.

Table 1 summarizes participant demographic information. Of note, participants across groups did not significantly differ in mean age, sex, handedness, race/ethnicity, or Hollingshead SES. However, the group with DS had significantly lower IQ scores, as expected.

**Table 1:** Participant demographics for Down syndrome and typically-developing control groups.

	Down syndrome (*n* = 19)			Control (*n* = 33)			Stat. significance
	*M*	*SD*	Range	*M*	*SD*	Range	
Age	16.53	4.99	7–23	17.53	5.15	9–24	n.s.
IQ	53.21	13.19	24–77	116.30	13.37	85–151	*t*(50) = − 16.46, *p* < 0.001
Verbal IQ	54.42	14.97	30–71	117.12	13.51	82–156	*t*(50) = − 15.5, *p* < 0.001
Nonverbal IQ	56.42	16.00	24–86	112.64	15.58	81–151	*t*(50) = − 12.41, *p* < 0.001
Hollingshead SES^a^	41.68	15.69	20–63	37.31	20.77	20–115	n.s.

	*n*	%		*n*	%		Stat. significance
Male,* n* (%)	6	32		15	45		n.s.
Right hand,* n* (%)^b^	12	63		25	76		n.s.
White, Non-hispanic, *n* (%)	15	79		22	67		n.s.

^a^Down syndrome, *n* = 19; Control, *n* = 29.

^b^Down syndrome, *n* = 18; Control, *n* = 33.

### Image acquisition

Structural and functional MRI data were acquired for participants on the same 3-Tesla General Electric Scanner using an 8-channel head coil. High-resolution (0.94 × 0.94 × 1.22 mm) T1-weighted anatomical ASSET-calibrated magnetization prepared rapid acquisition gradient echo (MPRAGE) images were acquired (128 axial slices; 224 × 224 acquisition matrix; flip angle = 12°; field of view = 240 mm). Next, gradient-echo echo-planar resting-state scans were collected for each participant. Resting-state scans lasted 5 min and 15 s (126 consecutive volumes with whole-brain coverage, repetition time = 2500 ms, echo time = 27 ms, 90° flip angle, 44 contiguous interleaved oblique slices per volume aligned to the AC-PC line, 2.8 mm slice thickness, 22 cm field of view, 64 × 64 acquisition matrix, 3.438 mm × 3.438 mm × 2.8 mm voxel size). Participants were instructed to lie quietly and fix their gaze on a central cross. Cardiac and respiratory signals were recorded during resting-state scans for image preprocessing.

### fMRI pre-processing

The Analysis of Functional NeuroImages (AFNI^44^) software package was used to process Echo-planar images (EPIs). The first three EPI volumes were first removed from each scan. Then, AFNI’s 3dDespike was applied to reduce any large deviations in voxel-wise signals. Next, EPI volumes were slice-time corrected to slice-time 0 and co-registered with the respective anatomical scans (MPRAGEs). Blurring was then applied using a 6 mm isotropic full-width at half-maximum Gaussian kernel and rescaled to percent signal change by normalizing each voxel’s mean blood-oxygen-level-dependent (BOLD) signal intensity. Lastly, scans were resampled to 3.0 mm^3^ voxels and transformed into standard Talairach and Tournoux^45^ space prior to group comparisons.

AFNI’s ANATICOR nuisance regression procedure was applied to the EPI data to regress nuisance artifacts (^46,47^; see also^48^). First, MPRAGE scans were segmented using FreeSurfer^49^. Next, ventricle and white matter masks were created, resampled to the EPI resolution, and eroded by 1 voxel to prevent partial volume effects with gray matter. Prior to spatial smoothing, a single average nuisance time series for the ventricles was calculated using the EPI data. As including a localized average white matter signal has been demonstrated to reduce dependence of functional connectivity on transient motion (e.g.^46^), we also included a localized average of white matter centered on each voxel and averaged within a 15 mm-radius sphere. Five respiration volume per time (RVT^50^) and 8 Retroicor^51^ regressors (4 respiratory and 4 cardiac) were generated using respiration and cardiac data collected during the scan. Additionally, the first three principal component time series extracted from a combined ventricle and white matter mask were calculated following the aCompCor denoising strategy (e.g.^52^; see also^48,53^). Nuisance variables were also detrended with fourth-order polynomials prior to least-squares model-fitting to every voxel’s time series. Thus, the full nuisance regression model included 6 motion parameters, an average ventricle time series, a localized white matter time series, eight Retroicor time series (4 cardiac, 4 respiration), 5 RVT time series, 3 principal component time series, and a fourth-order polynomial baseline model. The best fit time series of this nuisance model was then subtracted from the full, volume-registered time series to create residual time series for each participant.

### Motion assessment

Participant motion was evaluated with AFNI’s @1dDiffMag (comparable to mean frame-wise displacement in units of mm/TR). Groups differed significantly in motion (DS: *M* = 0.14, *SD* = 0.08, range 0.04–0.30; TD: *M* = 0.06, *SD* = 0.03, range 0.01–0.19; *t*(22) = 4.30, *p* < 0.001). Thus, all analyses were completed with motion covaried. Additionally, to assure that group differences in motion were not driving study findings, analyses were completed in a motion-matched DS and TD subsample. Results replicated those found for the whole sample when motion was covaried (see Results in Supplementary Figs. S1–S4).

### Analyses

Analyses were performed using AFNI^44^ and MATLAB. Mean functional “connectedness” for each participant was calculated (Pearson’s r) in every gray matter voxel (e.g.^54–56^). The mean correlation between each voxel with every other gray matter voxel in the participant’s brain mask was then stored back in the voxel (AFNI’s @3dTcorrMap). Connectedness values were then Fisher’s *z*-transformed to generate normally-distributed values. Next, group mean connectedness was compared between the DS and TD groups using two-sample *t*-tests (two-tailed; AFNI’s 3dttest ++) covaried for motion in each voxel in Talairach coordinates. To identify regions of interest (ROIs), results of the two-sample *t*-tests were then thresholded below false discovery rate (FDR)-corrected levels (*p* < 0.0001, *q* < 0.05) and a minimum requirement of 10 voxels was applied to define ROIs.

Subsequently, to examine whether differences in connectivity were network-specific, pairwise correlations using extracted average time series were calculated among each ROI-to-ROI pair for DS vs. TD participants using masks created for the ROIs that survived correction and which replicated in a motion-matched subsample. The ROI-ROI correlation matrices were normalized using Fisher’s *z* transformation and thresholded below FDR-corrected levels (*q* < 0.05).

To assess whether connectedness was related to selectivity in the DS group, we calculated network selectivity relative to the Yeo^33^ 17-network parcellation for both groups (selectivity = average within-network functional connectivity minus average between-network connectivity). The resulting selectivity values were then compared between groups. We conducted three ANCOVAs that included within-network connectivity, between-network connectivity, and network selectivity as the within-subjects factors, motion as the covariate, and group as the between-subjects factor. Additionally, we conducted an independent samples *t*-test on network selectivity by group. We then conducted a partial correlation between ROI-to-ROI mean connectivity and network selectivity, adjusted for motion.

Exploratory correlations with behavior using IQ (Full-scale, Verbal, and Performance) were conducted within the DS group. The ROI-ROI pairwise correlation values (a total of 18 × 17/2 = 153 for each participant) were first reduced to 18 values per participant using principal component analysis (the first 18 PCs) determined by the number of non-zero eigenvalues. These values for each participant were then entered as predictors in leave-one-out cross-validation regression analyses, with the behavioral measure as the dependent variable. For each iteration, one participant was left out and the regression model fitted using ridge regression [ridge parameter K = 5 using the function *ridge()* in MATLAB]. The fit parameters (without the y-intercept) were then applied to data from the left-out participant, generating a prediction for the behavioral score. After iterating through all participants, the predicted behavioral scores were correlated with the actual scores, with Type-I error controlled through permutation testing (repeating the same analyses while shuffling the behavioral scores across participants for 10,000 iterations).

## Results

### Question 1: Do youth with DS display aberrant whole-brain connectedness?

**Figure 1:**
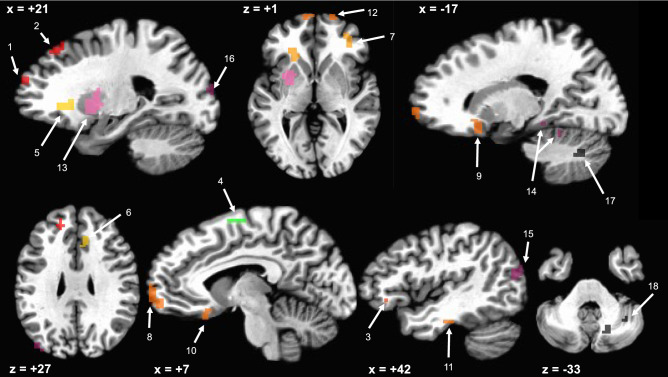
18 ROIs where functional connectivity is greater in participants with DS than TD controls, colored by the Yeo^33^ networks. Red = Default Mode; Green = Dorsal Attention; Yellow = Frontoparietal; Orange Limbic; Pink = Ventral Attention; Purple = Visual; Black = Cerebellar.

To answer our first question, whether youth with DS display differences in whole-brain connectedness relative to TD controls, we compared participants’ mean “connectedness” values across the two groups. By taking a whole-brain approach, we were also able to identify more localized brain regions with significantly higher connectedness in one group than the other by thresholding the t-test results below FDR-corrected-levels (*p* < 0.0001, *q* < 0.003) and setting a minimum ROI size criterion of 10 voxels. This resulted in 18 ROIs, all of which showed increased connectedness in the DS group relative to the TD group (Fig. 1, Table 2).

**Table 2:** 18 ROIs with group functional connectivity differences labeled by peak activation and Yeo^33^ network locations.

ROI	Location	Yeo^33^ network/cerebellar ROIs	X	Y	Z	Size (mm^3^)
1	L anterior frontal	DMN	− 16.5	− 52.5	+ 20.5	1539
2	L middle frontal	DMN	− 19.5	− 37.5	+ 47.5	1539
3	L inferior frontal	DMN	− 40.5	− 31.5	− 3.5	297
4	L superior frontal	Dorsal attention	− 10.5	+ 7.5	+ 59.5	459
5	L prefrontal	Frontoparietal	− 19.5	− 28.5	− 0.5	1296
6	R anterior Cingulate	Frontoparietal	+ 7.5	− 25.5	+ 29.5	648
7	R inferior frontal	Frontoparietal	+ 37.5	− 37.5	+ 2.5	567
8	L frontal pole	Limbic	− 7.5	− 61.5	− 3.5	1836
9	R insula	Limbic	+ 19.5	− 16.5	− 9.5	1161
10	L orbitofrontal	Limbic	− 4.5	− 22.5	− 15.5	864
11	L inferior temporal	Limbic	− 46.5	+ 16.5	− 21.5	486
12	R frontal pole	Limbic	+ 19.5	− 61.5	+ 2.5	297
13	L ventral frontal	Ventral attention	− 28.5	− 10.5	− 3.5	2862
14	R fusiform	Visual	+ 22.5	+ 34.5	− 15.5	2646
15	L TPO junction	Visual	− 46.5	+ 73.5	+ 17.5	1188
16	L middle occipital	Visual	− 22.5	+ 91.5	+ 14.5	567
17	R medial cerebellum	Cerebellar	+ 19.5	+ 61.5	− 33.5	405
18	R lateral cerebellum	Cerebellar	+ 37.5	+ 52.5	− 36.5	297

### Question 2: Is increased connectivity in youth with DS network-specific?

**Figure 2:**
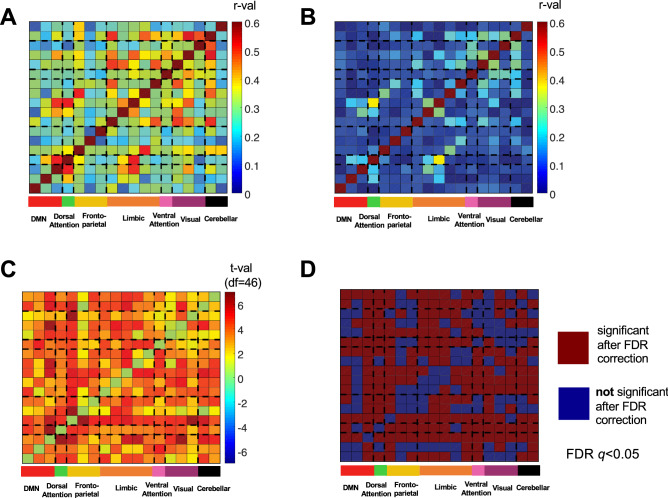
Connectivity matrices for the 18 ROI-ROI connections, each organized by the Yeo^33^ network structure. (**A**) Connectivity matrix (*r*-values) for participants with DS. (**B**) Connectivity matrix (*r*-values) for TD controls. (**C**) Connectivity differences for DS vs. TD participants t-test results (*t*-values) that replicated in a motion-matched subsample. (**D**) Significant (red) and non-significant (blue) connectivity differences from (**C**) after FDR correction.

Next, we examined whether increased connectivity seen in DS was network-specific using a correlation matrix for the 18 ROIs organized by the Yeo^33^ 7-network parcellation. No distinct network patterns emerged, suggesting the over-connectedness in DS is diffuse and not network-specific (Fig. 2A). For comparison, a correlation matrix for the 18 ROIs was also created for the TD group (Fig. 2B). Next, *t*-value (Fig. 2C) and significance (Fig. 2D) matrices were created to display which ROI-ROI pairs were significantly over-connected in DS after FDR correction. Most ROI pairs displayed greater functional connectivity in the DS group and survived FDR correction (*q* < 0.05, *p* < 0.0413). Although motion was covaried in these analyses, we further selected motion-matched subsamples (12 participants with DS and 15 TD participants) to test that these connections also survived this control (*q* < 0.05, *p* < 0.0346). All of the pairs displayed in Fig. 2C survived the group tests both with the full sample (with motion covaried) and with the motion-matched subsamples (see also Fig. S1).

### Question 3: Is increased connectivity related to reduced selectivity in youth with DS?

**Figure 3:**
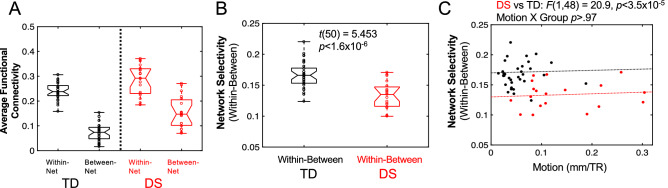
Network selectivity differences for participants with DS vs. TD controls. (**A**) Participants with DS display greater within- and between-network connectivity than TD controls. (**B**) Network selectivity (the difference between within-network connectivity and between-network connectivity) is reduced in participants with DS compared to TD controls. (**C**) Reduced selectivity in DS is not due to group differences in participant motion, indicated by a non-significant interaction between motion and group, *p* > 0.97.

We next examined whether connectivity is related to network selectivity in individuals with DS. In other words, is it possible DS is not just characterized by functional over-connectivity, but also reduced network integrity? First, we conducted a *t*-test comparing network selectivity by group, which revealed significantly reduced selectivity in youth with DS (Fig. 3B; *t*(50) = 5.453, *p* < 1.6 × 10^–6^). To ensure the group differences in network selectivity were not due to motion, we then conducted an ANCOVA, with group as the factor of primary interest, motion as a continuous nuisance factor, and network selectivity serving as the dependent variable. Notably for these analyses, selectivity was calculated over the entire voxel-level 17-network parcellation from Yeo^33^ and not solely using the ROIs that were significantly different between the groups. Results of the ANCOVA comparing network selectivity by group revealed a significant main effect of group [Fig. 3C; greater network selectivity in TD than DS: *F*(1,48) = 20.9, *p* < 3.5 × 10^–5^], but no main effect of motion [*F*(1, 48) = 0.2, *p* = 0.6564] and no significant interaction [*F*(1, 48) = 0.00126, *p* = 0.97], suggesting that reduced network selectivity in individuals with DS was not due to differences in motion (Fig. 3C). Next, we examined whether within- and between-network connectivity differences existed between groups using two ANCOVAs, with group as the independent variable, motion as a covariate, and either within- or between-network average functional connectivity as the dependent variables. Results of the within-network ANCOVA revealed a marginal effect of group [*F*(1, 48) = 3.86, *p* < 0.06], but no effect of motion [*F*(1, 48) = 1.83, *p* > 0.05] and no significant interaction [*F*(1, 48) = 0.16, *p* > 0.05], suggesting youth with DS have marginally higher within-network connectivity than TD peers that is not related to motion (Fig. 3A). Results of the ANCOVA for between-network connectivity revealed significant effects of group [*F*(1, 48) = 11.05, *p* < 0.002] and motion [*F*(1, 48) = 6.71, *p* = 0.01], but no significant interaction [*F*(1, 48) = 0.24, *p* > 0.05], suggesting youth with DS have significantly increased between-network connectivity relative to TD peers that is not due to motion (Fig. 3A).

**Figure 4:**
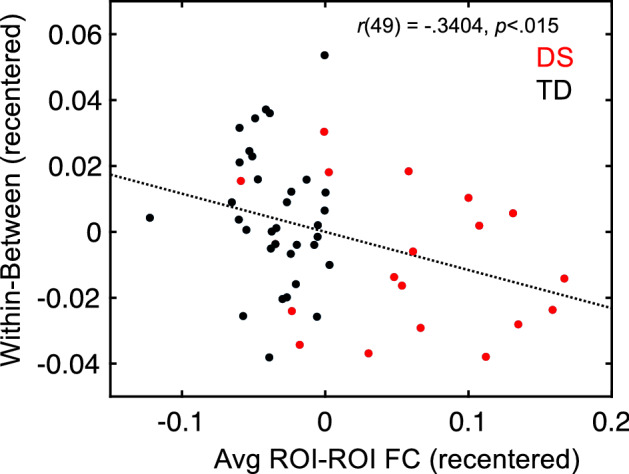
Increased ROI-ROI functional connectivity is significantly (*p* < 0.015) related to reduced network selectivity after partialling motion in the complete sample of participants.

We then evaluated the correlation between selectivity and the average connectivity level among the 18 ROIs, partialling motion. Although the correlation in the individual groups (DS, TD) failed to reach significance, the relationship between selectivity (within-between) and average ROI-ROI functional connectivity for both groups combined was significant after partialling motion [*r*(49) =  − 0.3404, *p* < 0.015; Fig. 4]. Taken together, these results suggest over-connectivity in youth with DS is characterized by increased between-network connectivity alongside reduced network selectivity, an effect that is not explained by motion.

### Exploratory behavioral analyses: Is increased functional connectivity related to behavior in youth with DS?

**Figure 5:**
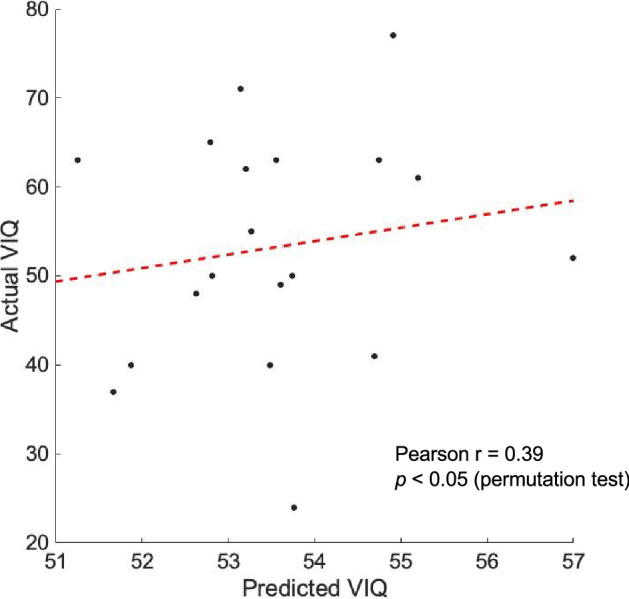
ROI-ROI correlation measures from participants with DS significantly predicted their VIQ scores by permutation testing using a leave-one-subject-out cross-validation approach (*p* < 0.05).

Finally, we conducted exploratory behavioral analyses to examine whether connectivity among regions showing increased connectivity in DS were predictive in DS of VIQ, PIQ, or FSIQ, all three of which are markedly impaired in this group (Table 1). The ROI-ROI correlation measures for participants with DS were used as predictors in a regression with VIQ, PIQ, and FSIQ as dependent measures in separate analyses. Participants’ ROI-ROI correlation values underwent data reduction using principal components analysis to arrive at 18 predictor values per participant. Ridge regression models were fit using leave-one-subject-out cross-validation, with each participant with DS left out once and the model parameters used to predict the behavioral score in each left-out participant (see “Methods” section for full details). Only VIQ was found to be significantly predicted using increased ROI-ROI connectivity values in the DS group [*r* = 0.39, *p* < 0.05 (uncorrected) by permutation testing over 10,000 iterations; Fig. 5]. While exploratory and preliminary, these results suggest that connectivity among these regions in individuals with DS may be related to verbal abilities.

## Discussion

The current study builds upon and adds to the existing literature examining differences in functional connectivity in young people with DS. Past studies on individuals with DS have yielded conflicting results, with some studies suggesting largely increased connectivity^28^ and others yielding a mix of both increased and decreased connectivity by region or network (e.g.^27,29^). Methodological decisions (i.e., analyzing group differences at the network- versus voxel-level, implementing global signal regression, or exposing participants to stimuli) may underlie discrepant findings in prior resting-state studies. Thus, the current study aimed to elucidate authentic resting-state functional connectivity in a sample of youth with DS using a data-driven approach and a traditional resting-state paradigm while implementing thorough controls for motion. Furthermore, to our knowledge, this study is the first that has examined the relationship between network connectivity and selectivity in youth with DS. Lastly, we explored potential behavioral correlates (i.e., IQ) of resting-state functional connectivity differences in youth with DS.

With key methodological factors carefully controlled, the current study revealed youth with DS display increased functional connectivity at rest across the whole brain relative to TD peers. Of note, this increased connectivity was not limited to select network pairs when organized by the Yeo^33^ 7-network parcellation. Rather, youth with DS were shown to have more highly connected brain activity throughout the whole brain. These findings are largely in line with Anderson’s^28^ results, which utilized similar data processing approaches (i.e., did not implement global signal regression). In contrast with Anderson’s^28^ study, we found no evidence of reduced connectivity in youth with DS, whereas results of their ROI-ROI correlation analyses revealed youth with DS displayed weaker connectivity compared to TD controls in a few longer-range ROI–ROI connections. Some possible explanations for this discrepancy in findings are the type of analyses performed (voxel-wise vs. ROI-ROI), the stimuli presented (traditional rest paradigm vs. cartoon-viewing), or sample demographic differences (i.e., *M* age = 16.60 years in the current study vs. *M* age = 20.2 years in Anderson’s study). In contrast with Anderson’s^28^ findings and the results of the current study, Pujol^27^ documented a mix of increased and decreased connectivity by region in youth with DS. One possible reason for this discrepancy is that Pujol^27^ implemented global signal regression. Indeed, a follow-up analysis of our data revealed that global signal regression resulted in finding reduced, rather than increased, connectivity in some regions, thus supporting the possibility that data processing approaches could be contributing to the discrepant findings (see Supplementary Fig. S5).

Regions showing increased functional connectivity in the DS group were highly consistent with findings from structural neuroimaging as well as with the neurocognitive profile of DS. In particular, a majority of regions identified (11 of 18 ROIs) were frontal regions, which is consistent with studies showing structural abnormalities in the frontal lobe^9,12^, executive functioning deficits characteristic of this population^3–5^, and prior resting-state study findings^27,28^. Additionally, a majority of the neocortical regions that showed increased connectivity (11 of 16 ROIs) were in the left hemisphere and the two cerebellar ROIs identified were in the right hemisphere. This pattern aligns with a profile of left-lateralized neocortical and right-lateralized cerebellar atypicalities, consistent with the prominent language impairments associated with DS^2^. Lastly, consistent with the findings from a recent, diffusion-driven tensor-based morphometry investigation^19^, the prominent involvement of regions not only in the frontal lobes but also the cerebellum highlight the potential importance of frontal-cerebellar networks in understanding the neural underpinnings of the DS cognitive phenotype. However, future research in which functional and structural findings are directly aligned would be more informative with respect to the extent of spatial overlap and convergence of physiological and anatomical phenotypes.

Turning now to the network selectivity findings, this study provides novel insights into our understanding of the associations between increased connectivity and reduced selectivity in youth with DS. Network selectivity (strengthened within-network connections and reduced between-network connections) underlies typical cognitive development and is observable at a young age^57–59^. Additionally, altered resting-state network integrity has been demonstrated to be linked to clinical outcomes (e.g., cognitive decline in older adults^60,61^). As individuals with DS experience precocious neurodegeneration^6^ and as network integrity degrades in healthy aging^61^, we sought to document whether reduced network integrity is detectable from a young age, prior to the onset of dementia. Further, we were interested in whether reduced network selectivity may be associated with alterations seen in whole-brain connectedness in individuals with DS. In particular, we sought to evaluate if over-connectivity in DS occurs alongside typically-developing network differentiation, or alternatively, if over-connectivity could be linked to reduced network selectivity. Our results were consistent with the latter—that is, youth with DS demonstrated reduced network selectivity relative to TD peers. Moreover, we documented that network selectivity was related to average connectivity level in the complete sample of DS and TD participants.

Taken together, our study’s findings suggest that reduced network selectivity is related to increased connectivity in youth with DS. Moreover, they provide the first evidence that altered network integrity is a part of the DS neuro*developmental* phenotype (i.e., this neural phenotype is present well before the onset of neurodegeneration). We hope that this finding will spur future investigations into the contribution of this earlier developmental state to the unfolding of later neurodegeneration in DS. In particular, future research may benefit from examining whether heterogeneity in the degree of network selectivity observed among young people with DS predicts variability observed later in the timing and occurrence of precocious-onset dementia.

In addition to the implications for understanding risk for later dementia in DS, the current study’s findings may also have broad relevance to understanding the neural underpinnings of neurodevelopmental disorders more generally. Although network selectivity alterations have been linked to cognitive outcomes in other populations, such as aging adults^60,61^, selectivity alterations have not been examined in populations with neurodevelopmental disorders. More specifically, many studies have examined within- and/or between-network connectivity differences in isolation in neurodevelopmental populations (e.g., autism spectrum disorder^62,63^; attention-deficit hyperactivity disorder^64^; schizophrenia^65,66^; bipolar disorder^53^); however, none, to our knowledge, have examined either the relationship between these measures, nor the relationship between selectivity and the degree of under- or overconnectivity.

As we only included a typically-developing control group in the current study, we cannot speak to whether reduced network selectivity (i.e., greater between- than within-network connectivity) is unique to individuals with DS or if reduced selectivity may be a neural phenotype associated with neurodevelopmental disorders characterized by cognitive impairment more generally. Moreover, given our cross-sectional study design, we are not able to speak to whether reduced network selectivity in DS is a relatively constant feature of the DS neural phenotype or whether it emerges in later childhood or adolescence. Future research should therefore examine this phenotype longitudinally to determine whether reduced network selectivity may be predictive of clinical outcomes in individuals with DS and other neurodevelopmental populations.

Although exploratory in nature due to our small sample size, we also examined potential behavioral correlates of observed connectivity differences. Research from other clinical populations suggests that investigating relations between behavior and brain findings may prove fruitful for prognostication. For example, resting-state abnormalities have been used to obtain prognostic information such as severity of disease in other neurodevelopmental disorders (i.e., schizophrenia^67^) as well as disease states in neurodegenerative disorders, such as Alzheimer’s disease (e.g.^68–70^). As verbal, performance, and full-scale IQ are impaired in individuals with DS, with individuals displaying declining IQ scores with age^71,72^, the results of these analyses may be informative not only for predicting end states (i.e., IQ at one point in time) but also atypical trajectories of cognitive development (i.e., how IQ changes over time) in a dynamic neurodevelopmental disorder like DS. Results of our behavioral analyses suggested that regional over-connectivity in youth with DS is associated with cognitive differences. Although the exact nature of the brain-behavior findings differ from Anderson’s^28^ findings, both studies suggest meaningful relationships between aspects of neural connectivity and cognitive outcomes. Future studies should examine whether connectivity differences are associated with individual differences in cognition in other clinical conditions associated with intellectual disability to determine whether this relation is syndrome-specific or more generally related to atypical neurodevelopment. Moreover, longitudinal research is needed to test the hypothesis that regional over-connectivity is not only related to cognition at one point in time but may also be predictive of changing cognitive abilities over development.

The current study has several notable strengths. First, we implemented standard data collection (i.e., the use of an eyes-open rest-state design) and preprocessing procedures (i.e., we did not implement global signal regression). As greater participant motion has been documented to be associated with functional connectivity^73^, we took great care to ensure that our findings were not due to differences in participant motion. Specifically, we included covariates for participant motion in our primary analyses, and we found that motion did not significantly impact any of our results. We also replicated our findings using motion-matched subsamples for which motion was further covaried. Another strength of the current study is that we recruited a young sample of individuals with DS well before the potential threat of Alzheimer’s disease could cloud study findings. Additionally, to our knowledge this is the first study to examine network selectivity and its relationship to over-connectivity in DS. Importantly, network selectivity differences in DS may be predictive of clinical outcomes such as cognitive decline. Finally, the current study is one of the few to demonstrate a link (though preliminary) between brain and behavior in a DS cohort.

Despite these strengths, one major limitation of the current study is the relatively small sample of participants with DS. However, our sample size (*n* = 19) was similar to or greater than the sample size of existing resting-state studies. For example, Anderson’s^28^ study included 15 adolescents and adults with DS, Pujol’s^27^ study included 20 adults with DS, and Vega’s^29^ study included just 10 adults with DS. Future research would benefit from larger samples in order to examine brain-behavior relations with greater confidence and to extend the cognitive phenotypes to include other domains that are relevant to DS, including executive function, language, and memory. Moreover, future studies should investigate connectivity and selectivity differences in individuals with DS at various stages of development through adulthood and extend functional connectivity research in DS to include a longitudinal study design. Finally, larger samples are needed to examine brain-behavior correlations in DS. Specifically, our exploratory analysis identifying links between verbal IQ and connectivity in DS needs to be replicated with a larger sample in order to confirm these findings. In addition, arranging for all participants to complete the same verbal IQ test in future studies would strengthen our confidence in the current study’s verbal IQ and connectivity findings.

Despite these limitations, the current findings speak to the discrepancies in the extant literature regarding resting-state connectivity in youth with DS by demonstrating that widespread increased connectivity in this population is related to reduced network selectivity. Furthermore, results of this study suggest that resting-state connectivity may be associated with verbal abilities in youth with DS. This study has significant clinical implications, as it suggests that reduced brain network selectivity and over-connectivity may be intervention targets for this population.

## Supplementary Information

Supplementary Information.

## Data Availability

Data available upon request through protocol PI, Armin Raznahan, MD, PhD. Contact corresponding author (N.R.L) with inquiries.
