# Supplementary material for: Youth with Down syndrome display widespread increased functional connectivity during rest
Source: Sci Rep. 2022 Jun 14;12:9836. doi: 10.1038/s41598-022-13437-1 (PMC9198034; doi:10.1038/s41598-022-13437-1)
Supplement: Supplementary file 1 — Supplementary Information. [file 41598_2022_13437_MOESM1_ESM.docx]

**Supplemental Information**

**Methods**

In total, 54 participants with DS initially enrolled in the larger study and six dropped out prior to completing MRI scans (due to family overcommitment or illness). Of the remaining 48, three participants with DS did not complete any scanning (structural or functional) due to inability to complete mock scanning procedures. Forty-five participants completed some aspect of scanning without sedation. Of these 45 participants, 19 provided usable resting-state fMRI data. Reasons for exclusion of the 26 participants for whom some aspect of scanning was attempted included non-compliance (resulting in no scan data being obtained) or excessive movement making the scans unusable. To examine group differences for those with (*n*=19) and without usable (or any) resting-state data (*n*=29), t-tests and chi-squares were used and the following variables were contrasted: age, IQ, and sex (Table S1). Of note, participants with and without usable scan data did not significantly differ in IQ scores. The group of participants with usable scan data was significantly older and contained a higher percentage of females than the group of participants without usable scan data. See Table S1 for details.

| **Table S1. *Group differences for participants with DS with and without usable (or any) resting-state data*** | | | | | |
| --- | --- | --- | --- | --- | --- |
|  | **With Usable Data (*n*=19)** | | **Without Usable Data (*n*=29)** | |  |
|  | ***M*** | ***SD*** | ***M*** | ***SD*** | **Stat. significance** |
| **Age** | 16.60 | 4.96 | 11.79 | 5.36 | *t*(46) = -3.13, *p* < 0.01 |
| **IQ** | 53.21 | 13.19 | 50.63 | 17.22 | n.s. |
| **Verbal IQ^1^** | 54.42 | 14.97 | 49.36 | 19.05 | n.s. |
| **Nonverbal IQ^1^** | 56.42 | 16.00 | 53.88 | 17.96 | n.s. |
|  | ***n*** | **%** | ***n*** | **%** | **Stat. significance** |
| **Male, *n* (%)** | 6 | 32 | 21 | 72 | *X2*(1, *N* = 48) = 7.78, *p* < 0.01 |
| ^1^Four of the 29 participants in the group without useable data did not have IQ data available to compare. Thus, sample size for this analysis was 25. | | | | | |

**Figure S1.** *Connectivity matrices for the* *18 ROIs organized by the Yeo [1] network structure*

*that replicate across the full and motion-matched samples.* (A) shows the t-values of DS-TD for the motion-matched samples, partialling motion and age. (B) is the conjunction of FDR-corrected pairs (*q*<.05) which are detected in both the full and the motion-matched samples, having removed any pairs exhibiting Group X Motion interactions at *p*<.05 (uncorrected).  The significance matrix shows that most ROI pairs exhibit greater connectivity in participants with DS than in TD participants, with none showing the reverse pattern.  ROIs are organized by the Yeo [1] 7-network parcellation as in Table 2.


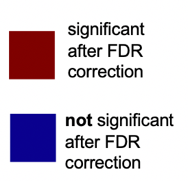

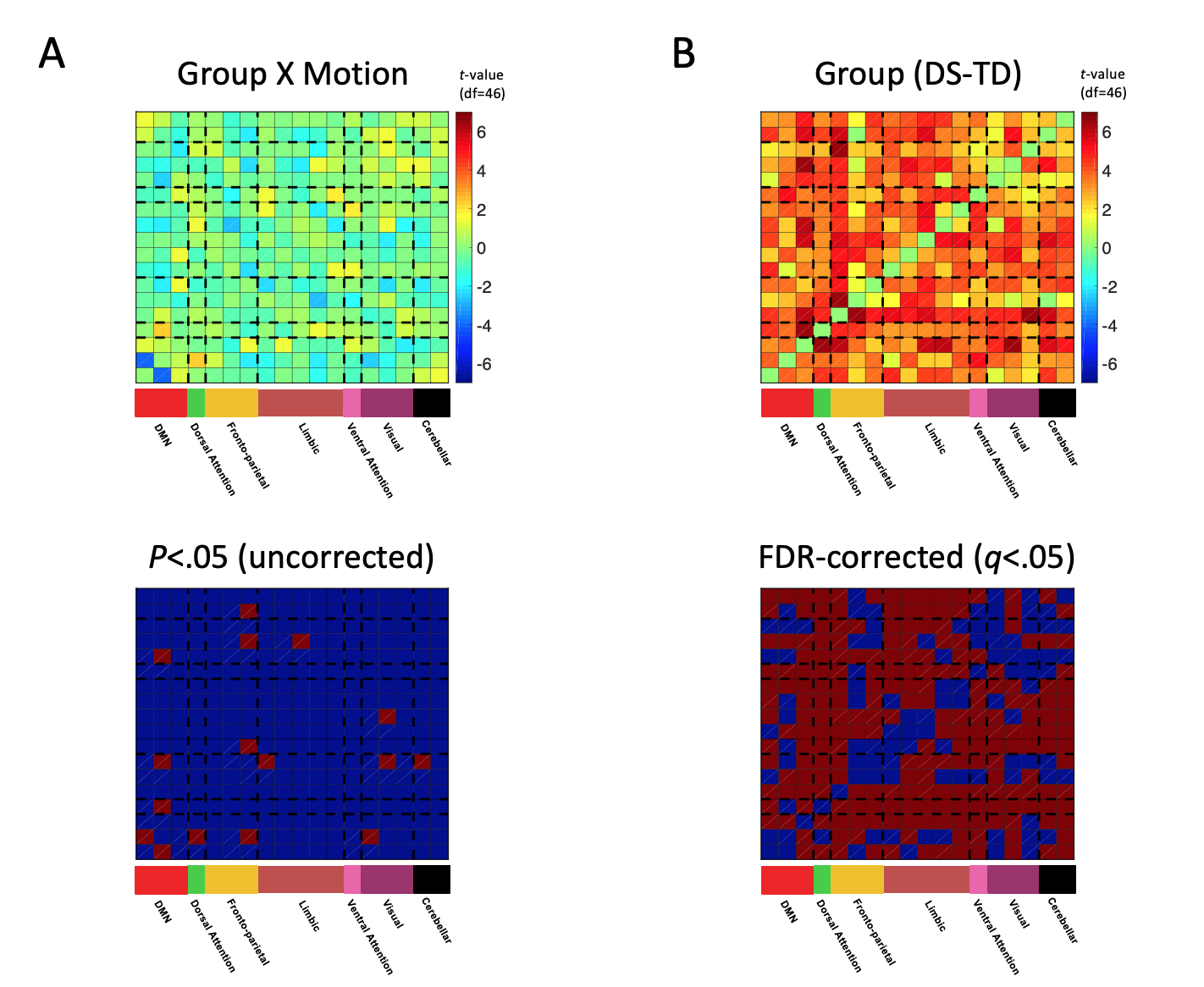


**Figure S2.** *ROI-ROI group x motion interaction tests and results excluding ROI pairs with a significant interaction.* (A) Group x motion interaction matrix for the 18 ROIs organized by the Yeo [1] 7-network parcellation (top) and significance matrix (bottom) at a *p*<.05 uncorrected level (8 ROI pairs). (B) The DS-TD correlation and significance matrices removing the eight ROI-ROI pairs displaying a significant group x motion interaction after FDR correction. The top matrix displays the new *t*-value matrix and the bottom displays the new significance matrix after FDR correction after removing these eight ROI pairs.

**Figure S3.** *Top 5 strongest group effects that fail to show group x motion effects.* The strongest ROI-ROI group effects (see correlation matrix, top left) display parallel slopes of motion (x-axes) on functional connectivity values (y-axes), demonstrating that the effects of motion and group are independent of one another.


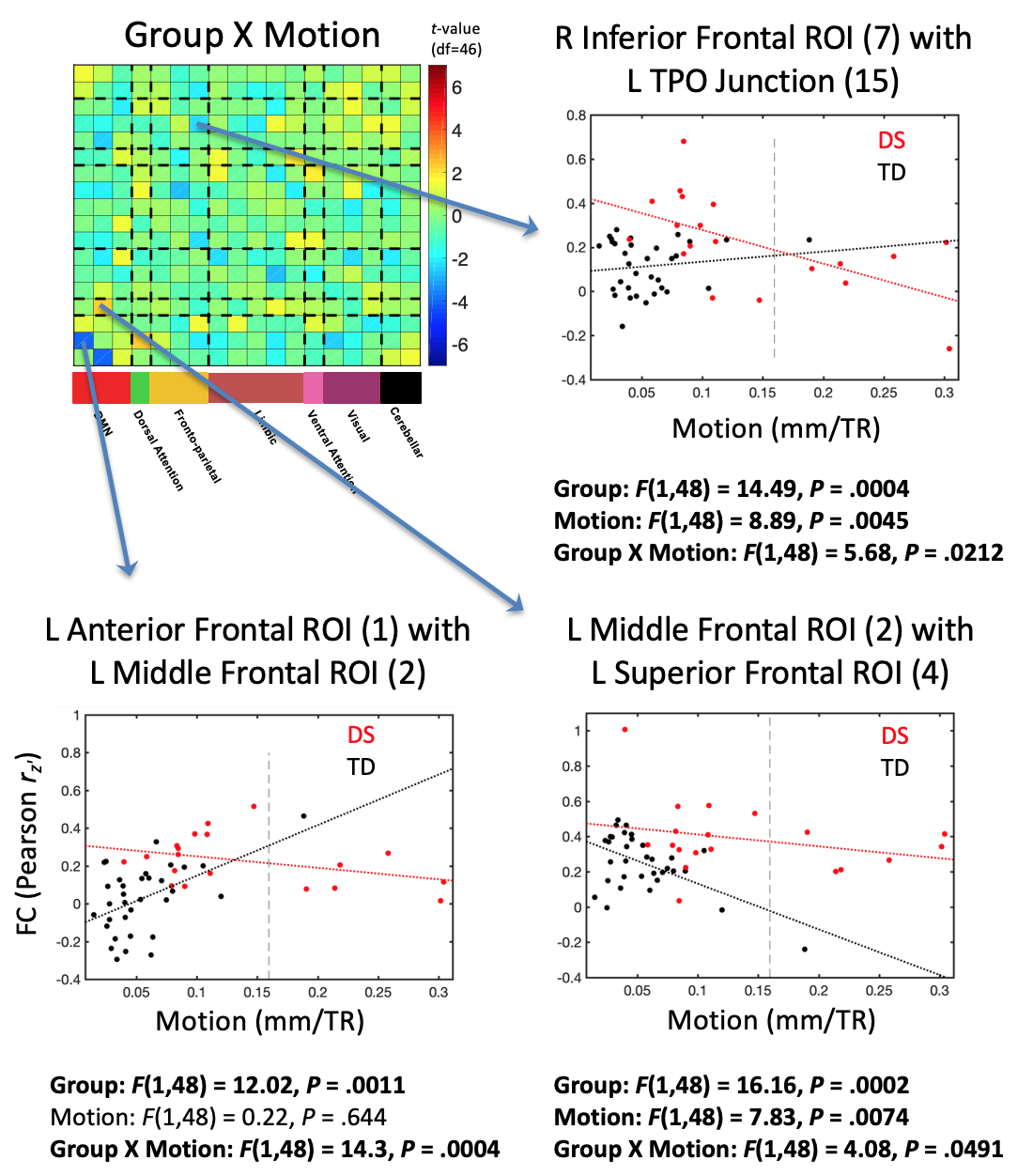


**Figure S4.** *Three example ROI-ROI pairs showing a significant group x motion interaction.* As eight of the 119 ROI-ROI pairs displayed a significant group x motion interaction effect (*p*<.05), although uncorrected for multiple comparisons, here we show three such instances. Cross-over interactions (top right, bottom left) suggest connectivity differences exhibited across groups may be influenced by motion levels in these ROI combinations. In the case of ROI2-ROI4 (bottom right), a significant effect of motion on connectivity in each group with non-parallel slopes suggests the value of the group difference may depend on the value of motion, with very little difference expected at motion values near zero.

**Figure S5.** *Effect of global signal regression on connectedness results.* Connectedness was compared without (no GSR) and with (GSR) global signal regression in the motion-matched subsamples (13 DS and 19 TD participants).  DS and TD groups did not differ in age, motion, or tSNR (*p*>.13 for all), and these variables were covaried in the connectedness analyses using AFNI's 3dMVM (multi-variate modeling). Unthresholded results are shown in the top panels, whereas thresholded results are shown in the bottom panels (*p*<.05, FDR *q*<.05 for the "no GSR" results, and *p*<.05, uncorrected for the "GSR" results). As previously reported by Pujol [2], underconnectivity rather than overconnectivity can be observed under GSR for the DS group in portions of the cingulate cortex (somatomotor) and the posterior insula. In contrast, overconnectivity is universally observed in the thresholded results without GSR. Note also that these differences cannot be easily explained by the effect of GSR on removing artifacts such as head motion, as these are matched and covaried in the group comparisons under both types of pre-processing.

**References**

1. Yeo, B.T., et al. The organization of the human cerebral cortex estimated by intrinsic functional connectivity*.* *J Neurophysiol*. **106**, 1125-65 (2011).

2. Pujol, J., et al. Anomalous brain functional connectivity contributing to poor adaptive behavior in Down syndrome*.* *Cortex*. **64**, 148-56 (2015).
